# Utility of salivary cortisol profile as a predictive biomarker in nurses’ turnover risk: a preliminary study

**DOI:** 10.1186/s40101-023-00349-w

**Published:** 2024-01-02

**Authors:** Shinya Yamaguchi, Tomoko Fujita, Shintaro Kato, Yuichi Yoshimitsu, Yoichi M. Ito, Rika Yano

**Affiliations:** 1 Department of Nursing, Teine Keijinkai Hospital, Sapporo, Japan; 2 Graduate School of Health Sciences, Hokkaido University, Sapporo, Japan; 3 NEC Solution Innovators, Ltd., Tokyo, Japan; 4 Data Science Center, Promotion Unit, Institute of Health Science Innovation for Medical Care, Hokkaido University Hospital, Sapporo, Japan; 5 Faculty of Health Sciences, Hokkaido University, Sapporo, Japan

**Keywords:** Personnel turnover, Nurses, Saliva, Cortisol, Fatigue, Burnout

## Abstract

**Background:**

Predicting nurse turnover risk is crucial due to the global nursing shortage; however, existing predictors, such as fatigue and burnout, lack objectivity. Salivary cortisol is a non-invasive marker of stress and fatigue, but its utility in predicting nurse turnover risk is unknown. We examined whether salivary cortisol profiles across three different day shifts in a month are predictors of the extent of nurses’ *reluctance to stay* in their current jobs.

**Methods:**

This preliminary longitudinal study followed forty female nurses who engaged in shift work at a university hospital for 3 months. Data at enrollment were collected including demographics, working conditions, chronic fatigue (the Japanese version of the Occupational Fatigue/Exhaustion Recovery Scale), and burnout (Japanese Burnout scale). Salivary cortisol was measured before the three different day shifts (after awakening) during the first month, and the means of these measurements were used as the cortisol profile. The extent of reluctance to stay was assessed using the numerical rating scale at 3 months.

**Results:**

Among the forty female nurses (mean [SD] age, 28.3 [5.1]), all completed follow-up and were included in the analysis. The cortisol profile was associated with the extent of reluctance to stay (*P* = 0.017), and this association was significant despite adjustments for chronic fatigue and burnout (*P* = 0.005). A multiple regression model with chronic fatigue, burnout, and job tenure explained 41.5% of the variation in reluctance to stay. When the cortisol profile was added to this model, the association of the cortisol profile was significant (*P* = 0.006) with an *R*^2^ of 0.529 (Δ*R*^2^ = 0.114).

**Conclusions:**

This preliminary study conducted in an actual clinical setting indicated the potential of the salivary cortisol profile across three different day shifts in a month to predict nurses’ reluctance to stay in their current jobs. The combination of subjective indicators and the cortisol profile would be useful in predicting nurses' turnover risk.

**Supplementary Information:**

The online version contains supplementary material available at 10.1186/s40101-023-00349-w.

## Background

The nursing profession accounts for more than 50% of the global shortage of healthcare workers [1]. This situation affects optimal staffing and the workload of nurses, resulting in decreased quality of care, increased patient mortality, and the deterioration of nurses’ occupational safety [2–5]. One solution to this nursing shortage is the prevention of turnover [6, 7], which requires detecting and addressing the early signs of nurse turnover.

Turnover and turnover intention are variables commonly measured in research related to nursing turnover [8–10]. These variables include employee-initiated turnover, resulting from personal decisions that nursing managers are unable to anticipate, and employer-initiated turnover, resulting from dismissals for employee performance-related issues. Hence, different situations require that nursing managers consider different countermeasures to mitigate the effects of turnover. Hom et al. [11] considered employees’ motivational states for staying or quitting, and posited that these mindsets can be explained by a combination of two aspects: desired employment status and perceived volitional control (whether quit or stay decisions are completely up to them or partially under external regulation). According to this theory, reluctant stayers are those who would prefer to leave but feel they cannot and they include those who can plan to leave their jobs following the expansion of their job search. Reluctant stayers may also have a low person-organization fit, which results in their expressing work avoidance behaviors (e.g., absences) or counterproductive workplace behaviors (e.g., sabotage) [11]. Nursing managers can identify at-risk nurses by recognizing reluctant stayers, and early signs of turnover, and provide interventions for turnover prevention.

Therefore, it is essential to identify predictors of nurses’ *reluctance to stay* in their current jobs. Chronic fatigue and burnout, as determined by individual perceptions, can predict nurses’ reluctance to stay, because these factors—along with an excessive workload and an inadequate work environment—are associated with turnover among nurses [12–14]. Chronic fatigue causes a reduction in nurses’ interest, motivation, and doubts over their ability to maintain their current work patterns [15]. Burnout is a result of prolonged work stress [16, 17], which induces negative emotions about the job [18, 19], and can increase reluctance to stay among nurses.

However, using chronic fatigue and burnout alone as predictors has limitations. The cycles of stress, fatigue, and burnout are insidious [20], and if individuals are not aware of them, the risk may remain undetected. Furthermore, the accuracy of subjective indicators is limited because they include uncontrollable biases, such as recall and confirmation bias.

Given these challenges, it is recommended the selection of physiological biomarkers for the evaluation of healthcare professionals [21]. Adaptation to stress involves a biological response (e.g., the hypothalamic–pituitary–adrenal [HPA] axis), and allostatic load (ie, the cumulative burden of chronic stress), as an abnormality of this response, is associated with pathological responses [22]. Cortisol is an indicator of the HPA axis and is suggested to be associated with work stress [23, 24], chronic fatigue [25, 26], and healthcare worker burnout [27]. Considering its application to the work setting, a convenient point-of-care device can be used to quickly and noninvasively measure cortisol using saliva samples [28]. A previous study of shift-working nurses identified salivary cortisol profiles across several different day shifts that were associated with chronic fatigue. Among cortisol profiles observed over 1 month, the profile of consistently low cortisol levels across two different day shifts was associated with higher chronic fatigue [29]. However, although the cortisol profiles are suggested to be associated with chronic fatigue among nurses [29], it remains unclear whether cortisol is a predictor of their reluctance to stay among nurses in actual clinical settings.

Therefore, in this preliminary study, we examined whether salivary the cortisol profile across three different day shifts in a month is a predictor of nurses’ reluctance to stay in their current jobs. Additionally, we explored the potential for better prediction of nurses’ reluctance to stay by combining the cortisol profile and subjective indicators. We hypothesized that the lower the cortisol profile, the higher the reluctance to stay among nurses. This study is the first step in examining the utility of the cortisol profile in the management of nursing turnover in actual work situations. The findings will provide preliminary suggestions for applying a new objective indicator to the management of turnover risk.

## Methods

### Study design and participants

This longitudinal study was conducted at a university hospital in Japan. The recruitment period for this study was from October 1, 2021, to March 31, 2022, and each participant was followed for 3 months. Participants included 40 female nurses aged 23–41 years working in a two-shift system (two 12-h shifts within 24 h) in a general ward. The gender and age of the participants were limited as factors affecting cortisol levels [29]. The exclusion criteria were as follows:

- New graduate nurses;
- Administrators;
- Nurses who regularly used sleeping pills, antipsychotics, antidepressants, steroids, and oral contraceptives;
- Pregnant nurses;
- Nurses on leave; and
- Nurses undergoing treatment for anemia, thyroid disease, diabetes, menstrual irregularities, insomnia, irregular heartbeat, insomnia, and autonomic nervous system disorders.

Posters outlining this study were distributed by the administrator to nurses in the target wards to recruit participants. This study was approved by the Ethics Review Committee of the Faculty of Health Sciences, Hokkaido University (reference No. 21-43) and was conducted in accordance with the Declaration of Helsinki. All participants provided written informed consent. Participants were offered a gratuity (Quo card) worth 12,000 yen.

### Study procedures

**Fig. 1:**
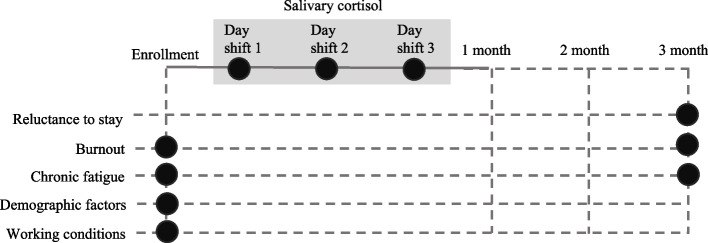
Study procedure. Black circles indicate measurements

Participants completed questionnaires regarding their demographics, working conditions, fatigue, and burnout. During the first month, they were asked to collect saliva samples upon awakening during three different day shifts. After 3 months, they reported the extent of their fatigue, burnout, and reluctance to stay (Fig. 1). In our study, the time interval between assessing predictors and the outcome evaluation was three months, which corresponds to a quarter term. This relatively short-term assessment of turnover risk has the following advantages in terms of nursing shortage: (1) early detection of turnover risk and prevention of problems becoming more severe, and (2) increased opportunity to identify challenges and dissatisfaction among nurses.

Considering the several influencing factors [30], the measurement of salivary cortisol at a single time point is not appropriate [29, 31] Two methods are used for multiple measurements: one is taking measurements multiple times on the same day (e.g., awakening response, diurnal variation) [32, 33] and the other is an inter-day trend [29, 34]. Our study considered inter-day trends to eliminate the burden of measurement and the influence of work conditions. Additionally, salivary cortisol measured in the early morning is associated with work stress and fatigue among nurses [23, 29, 31]. Considering the inter-day trend due to differences in work patterns and other factors, three different day shifts (one-time point more than in a previous study) were set as the measurement days [29].

### Reluctance to stay in the current job

Reluctance to stay was measured at three months using the question “How reluctant do you feel about continuing on your current job?” Participants responded to a rating scale ranging from 0 (*not at all*) to 10 (*extremely*). In related studies, nurses’ intention to leave is often assessed using a single item [14, 35, 36]. Additionally, our primary focus was to examine whether the cortisol profile is a predictor of reluctance to stay. In practice, the detailed background of nurses’ intention to leave can be ascertained through subsequent interviews by administrators after risk screening. Based on the above, we assessed only the extent of reluctance to stay using the aforementioned single question.

### Salivary cortisol

Participants gargled upon awakening, rested for 5 min, put on disposable gloves, and collected saliva samples with an oral fluid collector (OFC) swab (SOMA bioscience, Oxfordshire, United Kingdom). The OFC swab containing the saliva was placed into a 3-mL buffer solution. The buffer bottle containing the OFC swab was mixed for 2 min, then the OFC swab was removed from the bottle. Participants were asked in advance to avoid heavy exercise and alcohol consumption the day before saliva collection and to avoid eating, drinking, and brushing their teeth until the saliva was collected. Participants also reported their waking time, physical symptoms, and mood (e.g., irritability, depression) on the day of saliva collection, none of which were significantly correlated with each cortisol level. Saliva sample bottles were sealed in light-shielded bags, maintained at 37 °C or below, and submitted to the researcher upon arrival at work.

The saliva sample bottles were collected from the participants were kept frozen (up to 2 months) until analysis according to the instrument’s manual in order to maintain sample stability. The buffer solution containing saliva was placed in a cortisol lateral flow device (LFD) with three drops. A SOMA CUBE Reader (SOMA bioscience, Oxfordshire, United Kingdom) was placed on the LFD to measure cortisol concentrations. Cortisol levels measured using the SOMA CUBE reader [28, 37] and applying the same principle of measurement [38, 39] have revealed a positive correlation with the enzyme-linked immunosorbent assay (ELISA) method.

Each participant received an individual orientation session (lasting approximately 1 h) during which the detailed method of saliva sampling was explained. In this orientation, they received a manual which indicated the process and fully explained that saliva should be collected following 5 min of rest after awakening. The participants agreed to report any failure to adhere to these instructions to the researchers and understood that sampling dates would need to be rescheduled in such cases. No participant reported any violations of the sampling protocol, confirming their adherence to the procedure.

### Burnout

We used the verified and reliable Japanese Burnout Scale [40], developed in accordance with the Maslach Burnout Inventory to evaluate the participants’ burnout across three dimensions: emotional exhaustion, depersonalization, and decline in personal accomplishment. The inventory consists of 17 items on a five-point rating scale, ranging from 1 (never) to 5 (always). The average of the item scores included in each factor was used as the factor score. In each factor, a higher score indicates a stronger state of the condition.

### Chronic fatigue

We used the Japanese version of the 15-item Occupational Fatigue/Exhaustion Recovery Scale [41], and only 5 items of chronic fatigue were used in our analysis. The items are constructed on a seven-point Likert scale ranging from 0 (strongly disagree) to 6 (strongly agree). The standardized score of chronic fatigue (range 0–100) was calculated using the following formula: sum of the scores of the five items applicable to chronic fatigue divided by 30 and multiplied by 100. A higher standardized score indicates a stronger extent of chronic fatigue.

### Demographic factors and working conditions

A self-administered questionnaire was used to assess participants’ demographic factors and work conditions. The factors included age, job tenure, body mass index (kg/m^2^), marital status, childcare, family care role, alcohol consumption (drink or not drink), smoking habits (yes or no), and leisure time activities [42]. Working conditions included the number of night shifts worked per month; the total overtime hours per month (< 10 h, < 20 h, ≥ 20 h); the experience of less than 11 h of rest between shifts (a quick return) in the previous month; and perceived change in workload (i.e., decreased, unchanged, or increased vs previous month).

### Statistical analysis

The sample size was calculated using the G*Power version 3.1.9.7 (Universität Kiel). For hypothesis testing, we used a linear regression model with “reluctance to stay” as the objective variable and cortisol profile as the predictor, effect size (*f*^2^) = 0.20 (medium) [29], significance level = 0.05, power = 0.80, and a sample size of at least 42 would be sufficient.

Data were summarized using means (standard deviations [SD]) or frequencies (percentages). To normalize the distributions, all cortisol data were log-transformed before analysis log_10_ (X). For ease of interpretation, the results show the untransformed values. Correlations between variables were evaluated by Pearson correlation analysis. There was no consistent correlation between cortisol levels on each day shift and reluctance to stay (day shift 1: *r* = − 0.450, day shift 2: *r* = − 0.256, day shift 3: *r* = − 0.368). Because consistent levels across two different day shifts are associated with chronic fatigue in nurses [29], previous studies [43] have used the average cortisol level over two days. In contrast, this study used more time points for cortisol measurements, with an average of three measurements, to represent the cortisol profile.

Four separate linear regression models were performed with “reluctance to stay” as the objective variable. First, we assessed the association between the cortisol profile and reluctance to stay by a univariate regression model for our hypothesis (Model 1). Next, we adjusted for chronic fatigue and burnout during the enrollment of the study and examined the association of the cortisol profile (Model 2). Following this, a multiple regression model was performed with job tenure significantly associated with “reluctance to stay” (*r* = − 0.425, *P* = 0.006) and subjective indicators as explanatory variables (Model 3). The subjective indicators in Model 3, selected based on the lowest Akaike information criterion using the backward elimination method, were chronic fatigue and decline in personal accomplishment. Finally, Model 4, which added the cortisol profile to the explanatory variables, evaluated the association of the cortisol profile. In all models, we reported the coefficient of determination (*R*^*2*^) and the adjusted *R*^*2*^.

Statistical analysis was performed using JMP Pro software, version 16.1 (SAS Institute Inc., Cary, NC, USA), with *P* < 0.05 considered statistically significant.

## Results

### Participants characteristics

Among the 40 participants, none dropped out, and all were included in the analysis. Participants ages ranged from 23 to 41 years, with a mean (SD) of 5.3 (4.0) years of job tenure (Table 1). None had a smoking habit and 47.5% (19/40) had a drinking habit. Among the demographic and work conditions data, quick return in the month prior to study enrollment was significantly associated with the cortisol profile (see Additional file 1). Meanwhile, none of the data presented in Table 1, including quick return, were statistically significantly associated with the extent of *reluctance to stay* after three months.

**Table 1:** Participants’ demographics and working conditions at enrollment (*n* = 40)

	Mean (SD)/number (%)	Range
Age, mean (SD), years	28.3 (5.1)	23.0–41.0
Job tenure, mean (SD), years	5.3 (4.0)	1.0–18.0
Body mass index, mean (SD), kg/m^2^	19.6 (1.8)	16.0–25.0
Marital status, no. (%)		
Married or with a partner	7 (17.5%)	
Not married	33 (82.5%)	
Dependent, children, no. (%)		
Yes	1 (2.5%)	
No	39 (97.5%)	
Dependent, care role, no. (%)		
Yes	1 (2.5%)	
No	39 (97.5%)	
Drink alcohol, no. (%)		
Yes	19 (47.5%)	
No	21 (52.5%)	
Smoke cigarettes, no. (%)		
Yes	0 (0%)	
No	40 (100.0%)	
Exercise-oriented, no. (%)		
Yes	11 (27.5%)	
No	29 (72.5%)	
Sleep-oriented, no. (%)		
Yes	24 (60.0%)	
No	16 (40.0%)	
Activity-oriented, no. (%)		
Yes	10 (25.0%)	
No	30 (75.0%)	
Number of night shifts per month, mean (SD)	4.6 (0.9)	2.0–6.0
Overtime hours in the previous month, no. (%)		
< 10 h	11 (27.5%)	
< 20 h	17 (42.5%)	
≥ 20 h	12 (30.0%)	
Experience of quick return, no. (%)^, a^		
Yes	12 (30.0%)	
No	28 (70.0%)	
Change in workload, no. (%)		
Decreased	3 (7.5%)	
Not change	27 (67.5%)	
Increased	10 (25.0%)	

*SD* standard deviation

^a^Quick return refers to less than 11 h of rest between shifts

### Summary and correlations of key variables

Descriptive statistics for reluctance to stay, chronic fatigue, burnout, and cortisol profile are illustrated in Table 2. The means for both chronic fatigue and burnout were similar to previous studies based on large samples [41, 44]. Among the subjective indicators upon enrollment in the study, chronic fatigue (*r* = 0.565, *P* < 0.001), and emotional exhaustion (*r* = 0.565, *P* < 0.001), depersonalization (*r* = 0.360, *P* = 0.022), and decline in personal accomplishment (*r* = 0.463, *P* = 0.003) correlated moderately or more with reluctance to stay.

**Table 2:** Summary and Pearson correlations of key variables (*n* = 40)

	Mean (SD)	Range	1	2	3	4	5	6	7	8	9	10
1 Reluctance to stay (T1)	4.8 (2.0)	0-10		0.663	0.565	0.673	0.565	0.420	0.360	0.256	0.463	− 0.375
2 Chronic fatigue (T1)	52.8 (16.4)	0-100	< 0.001		0.796	0.801	0.709	0.483	0.525	0.228	0.439	− 0.250
3 Chronic fatigue (T0)	49.5 (18.2)	0-100	< 0.001	< 0.001		0.711	0.725	0.498	0.585	0.294	0.415	− 0.056
4 BOS-EE (T1)	3.4 (0.9)	1-5	< 0.001	< 0.001	< 0.001		0.846	0.597	0.639	0.145	0.335	− 0.341
5 BOS-EE (T0)	3.4 (0.9)	1-5	< 0.001	< 0.001	< 0.001	< 0.001		0.640	0.756	0.319	0.445	− 0.120
6 BOS-DP (T1)	1.9 (0.7)	1-5	0.007	0.002	0.001	< 0.001	< 0.001		0.819	0.263	0.222	− 0.160
7 BOS-DP (T0)	1.9 (0.7)	1-5	0.022	0.001	< 0.001	< 0.001	< 0.001	< 0.001		0.291	0.246	− 0.177
8 BOS-PA (T1)	3.7 (0.6)	1-5	0.111	0.157	0.065	0.373	0.045	0.100	0.069		0.775	0.295
9 BOS-PA (T0)	3.7 (0.6)	1-5	0.003	0.005	0.008	0.035	0.004	0.168	0.125	< 0.001		0.019
10 Cortisol profile, nM	6.6 (4.3)^a^	1-40	0.017	0.120	0.732	0.031	0.460	0.323	0.276	0.065	0.905	

Correlation coefficients appear on the top triangle, and *P* values of correlation coefficients appear on the bottom triangle

*Abbreviation*: *T0* at the enrollment of the study, *T1* after three months, *BOS* burnout, *EE* emotional exhaustion, *DP* depersonalization, *PA* decline in personal accomplishment, *SD* standard deviations

^a^ Cortisol profile (nM) values represent those without log transformation

### Association between the cortisol profile and nurses’ reluctance to stay

Table 3 shows the results of the multiple regression model examining the association of cortisol profile with nurses’ reluctance to stay. In Model 1, the cortisol profile was negatively associated with the extent of reluctance to stay at three months (*b* [unstandardized regression coefficient] = − 2.97; 95% CI, − 5.39 to − 0.56; *P* = 0.017). When adjusted for subjective indicators (Model 2), the cortisol profile was also negatively associated with reluctance to stay (*b* = − 2.83; 95% CI, − 4.74 to − 0.93; *P* = 0.005). In a stepwise multiple regression model that used chronic fatigue and decline in personal accomplishment and job tenure (Model 3), the significant predictor was chronic fatigue (*b* = 0.05; 95% CI, 0.02–0.08; *P* = 0.020), with *R*^2^ = 0.415. Chronic fatigue (*b* = 0.05; 95% CI, 0.02–0.08; *P* = 0.020) and cortisol profile (*b* = − 2.70; 95% CI, − 4.59 to − 0.82; *P* = 0.006) were significant predictors in Model 4. In this model, the *R*^2^ was 0.529, which increased from Model 3 (Δ*R*^*2*^ = 0.114).

**Table 3:** Association between reluctance to stay (outcome variable) and chronic fatigue, burnout, and cortisol profile (*n* = 40)

	Model 1			Model 2			Model 3			Model 4		
	*b* (95% CI)	*β*	*P* value	*b* (95% CI)	*β*	*P* value	*b* (95% CI)	*β*	*P* value	*b* (95% CI)	*β*	*P* value
Cortisol profile	− 2.97 (− 5.39, − 0.56)	− 0.38	0.017	− 2.83 (− 4.74, − 0.93)	− 0.36	0.005				− 2.70 (− 4.59, − 0.82)	− 0.34	0.006
Scale score (T0)												
Chronic fatigue				0.04 (0, 0.08)	0.32	0.070	0.05 (0.02, 0.08)	0.47	0.020	0.05 (0.02, 0.08)	0.44	0.020
BOS-EE				0.77 (− 0.24, 1.78)	0.34	0.130						
BOS-DP				− 0.64 (− 1.79, 0.51)	− 0.21	0.268						
BOS-PA				0.76 (− 0.11, 1.64)	0.24	0.086	0.30 (− 0.95, 1.55)	0.09	0.632	0.48 (− 0.66, 1.63)	0.15	0.399
Covariate												
Job tenure							− 0.13 (− 0.31, 0.05)	− 0.25	0.162	− 0.10 (− 0.27, 0.07)	− 0.20	0.229
*R*^2^	0.141		0.017	0.542		< 0.001	0.415		< 0.001	0.529		< 0.001
Adjusted *R*^2^	0.118			0.475			0.366			0.475		

*Abbreviation: T0* at the enrollment of the study, *BOS* burnout, *EE* emotional exhaustion, *DP* depersonalization, *PA* decline in personal accomplishment, *b* unstandardized regression coefficient, *CI* confidence interval, *β* standardized partial regression coefficient, *R*^2^ coefficient of determination

## Discussion

The management of nursing turnover risk has challenges related to a lack of objectivity. As the first step in overcoming this challenge, this study examined the cortisol profile’s potential and utility in predicting turnover risk among clinical nurses. Our study revealed that the cortisol profile in the first month was associated with the extent of nurses’ reluctance to stay after three months. The association of the cortisol profile was also significant in multiple regression models adjusted for subjective indicators and job tenure. These results support our hypothesis and extend the existing knowledge, such as the association between chronic fatigue and the cortisol profile across several day shifts among shift work nurses [29]. Therefore, the cortisol profile has potential applications in not only fatigue assessment but also in detecting reluctance to stay and the possible risk of nurse turnover.

One mechanism that explains the association between the cortisol profile and nurses’ reluctance to stay is the negative feedback response of the HPA axis to persistent stress [45]. Stress stimulates the release of cortisol, and this response returns to normal after the stress event has resolved; however, prolonged stress is considered to result in abnormal (i.e., low) cortisol secretion [46–48]. Cortisol secretion regulates energy production, metabolism, and mood and plays a role in coping with stress [49, 50]. Thus, low saliva cortisol levels over a period may be associated with the ability to cope adequately with further challenges and may affect nurses’ reluctance to stay.

In multiple regression models, the combination of subjective indicators and the cortisol profile better explained nurses’ reluctance to stay. These results highlight the importance of using both indicators, as relying on only one may only partially capture turnover risk. In particular, the cortisol profile has significance in predicting nurses’ turnover risk for which effective biomarkers have not been discovered. Previous studies have shown that burnout and other psychological factors only partially explain nurses’ turnover intentions (*R*^2^ = 39%) [13]. Additionally, the variation of reluctance to stay explained by subjective indicators and job tenure was limited (*R*^2^ = 42%). This limitation of sufficiently predicting turnover risk with only subjective indicators may be influenced by nurses' characteristics, such as their attitude toward social expectations and sense of duty as nurses [51]. Therefore, the cortisol profile can be a valuable objective indicator for predicting turnover risk among nurses.

Our findings have several implications for detecting nurse turnover risk effectively. First, self-monitoring using the cortisol profile can help prevent work stress from escalating into a turnover risk, such as reluctance to stay [52]. Traditionally, salivary cortisol measurement has only been possible using the ELISA method, which requires specialized knowledge and facilities. However, the SOMA CUBE reader is superior because it is a portable device that can be used in any location and is simple to operate. Given its easy, non-invasive, and immediate nature, the cortisol profile can be a self-assessment tool for nurses to assess their turnover risk. Based on our results, nurses might be able to use salivary cortisol samples monthly on three different day shifts. Second, the cortisol profile predicted nurses’ reluctance to stay after three months, providing a preparatory period for support and early intervention for high-risk individuals. Third, early signs of reluctance to stay must be screened with high sensitivity to address the turnover risk in the severe nursing shortage. Our findings suggest that nurses with high levels of reluctance to stay exhibit the physiological signs characterized by the cortisol profile or the psychological signs such as burnout and chronic fatigue. As an advantage of using both the cortisol profile and subjective indicators, concretely, even if the subjective indicators do not detect high-risk individuals, they may still be screened with cortisol and vice versa.

### Limitations

First, the sample was recruited from only one hospital in Japan, and participants’ characteristics were limited. As the study was conducted during the COVID-19 pandemic, nurses’ working conditions and the extent of chronic fatigue and burnout could deviate from the norm. However, since specific information, such as which participants had treated COVID-19 patients, was not collected, the pandemic’s impact could not be controlled for. Therefore, it is unclear whether our results can be applied to nurses with other characteristics or to other professions in different situations.

Second, salivary cortisol secretion can be affected by circadian rhythm changes due to irregular shift patterns [53], menstrual cycles, and various other personal factors. However, it was difficult to comprehensively collect and control for the numerous individual factors and diverse shift patterns in this clinical settings survey. Therefore, the influence of these factors on our results must be acknowledged. In order to mitigate the inter-day variation of salivary cortisol levels caused by the aforementioned factors, we employed the approach of calculating the average cortisol levels across three distinct daily shifts to create the cortisol profile. This allowed for smoothing out the inter-day variations in cortisol and showed long-term trends. It would also be somewhat robust to the effects of irregular shift patterns, menstrual cycle, and circadian rhythm changes.

Third, we did not measure the outcome (reluctance to stay) at study enrollment. We focused primarily on predicting the degree of reluctance to stay after three months, and the significance of the change in reluctance to stay was unclear. Additionally, there were some concerns about how other questions would be influenced or biased by measuring reluctance to stay at enrollment. Consequently, we could not examine the cross-sectional association between cortisol profile and reluctance to stay, nor could we describe the characteristics of reluctance to stay at enrollment.

Future studies that include a larger number of nurses and consider a variety of influencing factors could strengthen our findings and provide a threshold for the cortisol profile. Based on our preliminary findings, we contend that the practical implementation of this cortisol profile necessitates thorough validation through the collection of menstrual cycles and shift patterns, while also adjusting for their influences. The additional studies involving a larger population of nurses are imperative in order to comprehensively address the multitude of these factors. This would improve the utility of the cortisol profile and contribute to improved accuracy in screening for nursing turnover risk and reduced turnover rates.

## Conclusions

This preliminary study conducted in an actual clinical setting indicated the potential of the salivary cortisol profile across three different day shifts in a month to predict nurses’ reluctance to stay in their current jobs. It found that the cortisol profile is an objective predictor of nurses’ reluctance to stay independent of subjective indicators such as chronic fatigue and burnout. In addition, the cortisol profile in combination with subjective indicators may contribute to a better prediction of reluctance to stay. Therefore, combining self-monitoring of the salivary cortisol profile and surveillance with subjective indicators can be a valuable tool for predicting nurse turnover risk.

### Supplementary Information

**Additional file 1.**

## Data Availability

The datasets generated and/or analyzed during the current study are not publicly available due to ethical restrictions or confidentiality of participants but are available from the corresponding author on reasonable request.

## Abbreviations

BOS: Burnout
EE: Emotional exhaustion
DP: Depersonalization
PA: Decline in personal accomplishment
CI: Confidence interval
HPA: Hypothalamic–pituitary–adrenal
OFC: Oral fluid collector
LFD: Lateral flow device
SD: Standard deviations
