# Supplementary material for: Utility of salivary cortisol profile as a predictive biomarker in nurses’ turnover risk: a preliminary study
Source: J Physiol Anthropol. 2024 Jan 2;43:1. doi: 10.1186/s40101-023-00349-w (PMC10759393; doi:10.1186/s40101-023-00349-w)
Supplement: Supplementary file 1 — Additional file 1. [file 40101_2023_349_MOESM1_ESM.docx]

**Additional file 1.**

**Table 1. Correlation between the cortisol profile and demographics and working conditions**

|  | **Pearson's correlation coefficient (*r*)** | ***P* value*** |
| --- | --- | --- |
| Age | 0.115 | 0.480 |
| Job tenure | 0.064 | 0.694 |
| Body mass index | 0.022 | 0.893 |
| Number of night shifts per month | -0.180 | 0.267 |
|  | **Cortisol profile [nM], mean (SD)** | ***P* value^†^** |
| Marital status |  | 0.219 |
| Married or with a partner (n = 7) | 7.0 (8.3) |  |
| Not married (n = 33) | 6.5 (3.1) |  |
| Drink alcohol |  | 0.989 |
| Yes (n = 19) | 7.0 (5.4) |  |
| No (n = 21) | 6.2 (3.2) |  |
| Exercise-oriented |  | 0.422 |
| Yes (n = 11) | 6.4 (3.0) |  |
| No (n = 29) | 6.6 (4.0) |  |
| Sleep-oriented |  | 0.440 |
| Yes (n = 24) | 6.2 (3.5) |  |
| No (n = 16) | 7.0 (5.4) |  |
| Activity-oriented |  | 0.755 |
| Yes (n = 10) | 6.0 (2.2) |  |
| No (n = 30) | 6.7 (4.8) |  |
| Overtime hours in the previous month |  | 0.792 |
| < 10 h (n = 11) | 6.7 (4.0) |  |
| < 20 h (n = 17) | 7.2 (5.4) |  |
| ≧ 20 h (n = 12) | 5.5 (2.7) |  |
| Experience of quick return^a^ |  | 0.021 |
| Yes (n = 12) | 4.1 (1.4) |  |
| No (n = 28) | 7.6 (4.7) |  |
| Change in workload |  | 0.405 |
| Decreased (n = 3) | 4.5 (1.9) |  |
| Unchanged (n = 27) | 6.4 (4.8) |  |
| Increased (n = 10) | 7.6 (3.4) |  |

*SD* standard deviation.

^a^ Quick return refers to less than 11 hours of rest between shifts.

* Pearson's correlation analysis.

^†^Mann–Whitney U test or Kruskal-Wallis test.

Cortisol profile (nM) values represent those without log transformation.
